# Supplementary material for: Autonomic Function Recovery and Physical Activity Levels in Post-COVID-19 Young Adults after Immunization: An Observational Follow-Up Case-Control Study
Source: Int J Environ Res Public Health. 2023 Jan 27;20(3):2251. doi: 10.3390/ijerph20032251 (PMC9915325; doi:10.3390/ijerph20032251)
Supplement: Supplementary file 1 [file ijerph-20-02251-s001.zip › ijerph-2085283-supplementary.pdf]

Supplementary Materials

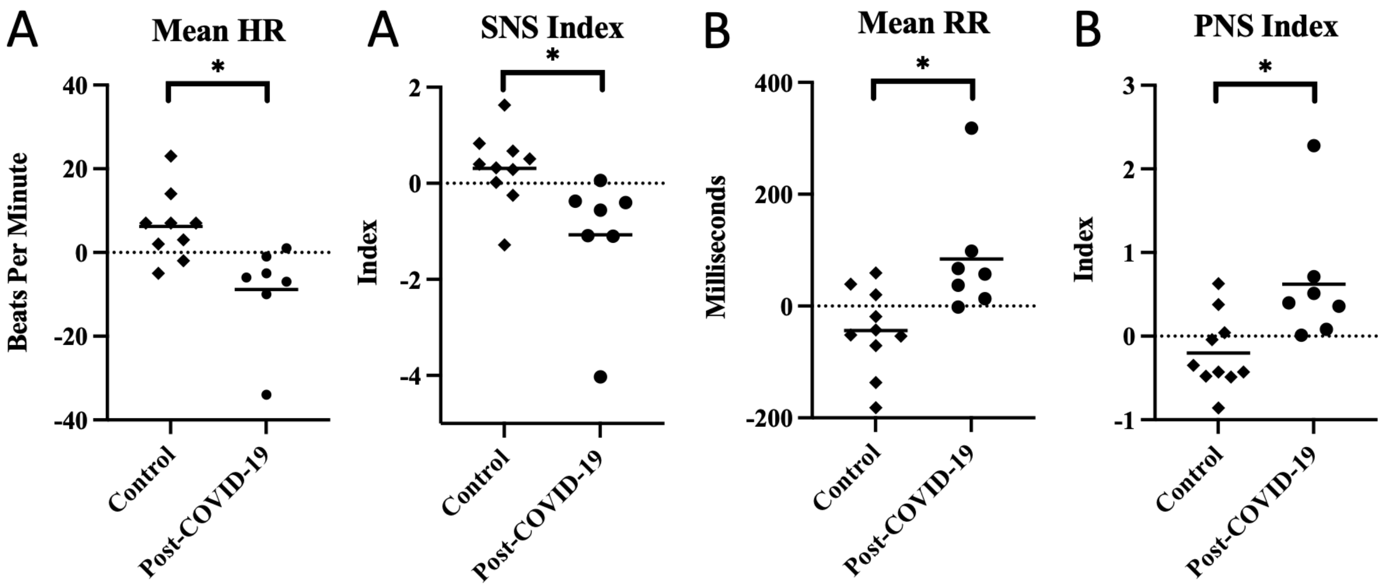

Figure S1. Scatterplot of HRV indexes for unpaired analysis according to group expressed as the mean difference between follow-up and baseline moments. (**A**) Sympathetic nervous system activity; (**B**) parasympathetic nervous system activity. * Statistical difference between groups; mean HR: average heart rate measured in beats per minute; SNS Index: sympathetic nervous system index; mean RR: time between.
